# FANCA facilitates G1/S cell cycle advancement, proliferation, migration and invasion in gastric cancer: FANCA’s role in G1/S cell cycle progression and beyond in GC

**DOI:** 10.3724/abbs.2024045

**Published:** 2024-04-29

**Authors:** Wei Wang, Shantanu Baral, Bin Liu, Qiannan Sun, Liuhua Wang, Jun Ren, Dong Tang, Daorong Wang

**Affiliations:** 1 The Yangzhou School of Clinical Medicine of Dalian Medical University Yangzhou 225001 China; 2 Northern Jiangsu People’s Hospital Affiliated to Yangzhou University Yangzhou 225001 China; 3 Northern Jiangsu People’s Hospital Yangzhou 225001 China; 4 General Surgery Institute of Yangzhou Yangzhou University Yangzhou 225001 China; 5 Yangzhou Key Laboratory of Basic and Clinical Transformation of Digestive and Metabolic Diseases Yangzhou 225001 China

**Keywords:** gastric cancer, FANCA, proliferation, migration, invasion, cell cycle

## Abstract

The present study explores the function of *FANCA* gene, a pivotal member of the Fanconi anaemia (FA) pathway crucial for preserving genomic stability and preventing cancer, particularly in the context of gastric cancer (GC). Using immunohistochemistry, quantitative real-time PCR, and western blot analysis, we evaluate FANCA mRNA and protein expressions in GC cell lines. The relationship between FANCA expression and clinicopathological characteristics is also explored. Various assays, including CCK8, colony formation, wound healing, and Transwell assays, are used to assess functional changes in cells associated with FANCA. Flow cytometry is utilized to evaluate alterations in the cell cycle resulted from *FANCA* knockdown and overexpression. Our findings show elevated FANCA expression in GC cell lines, with levels correlated with pathologic stage and lymphatic metastasis. *FANCA* knockdown impedes cell proliferation, migration, and invasion and induces G1/S phase cell cycle arrest. Conversely, *FANCA* overexpression stimulates cell proliferation, migration, and invasion. *In vivo* xenograft experiments confirm the promotional role of FANCA in GC tumor progression. Moreover, *FANCA* overexpression is associated with the activation of cell cycle. Collectively, our results suggest that FANCA drives malignant cell behaviors in GC through the cell cycle pathway, highlighting its potential as a therapeutic target for the treatment of GC.

## Introduction

Gastric cancer (GC), or stomach cancer, remains a challenging global health concern, demanding continued research and innovation for prevention, early detection, and treatment. This malignancy is associated with significant morbidity and mortality and ranks as the fifth most common cancer worldwide, with substantial regional variations in its incidence and outcomes [ 1, 2] . Emerging research is shedding light on the complexities of GC, including its genetic and molecular underpinnings, paving the way for novel approaches to deal with this formidable disease. The global distribution of GC is marked by notable disparities, with a disproportionately higher burden in specific regions, such as Eastern Asia and parts of South America, and a lower incidence in North America and Western Europe [3]. These geographic differences reflect a convergence of genetic, environmental, and lifestyle factors that contribute to disease initiation and progression. The role of *Helicobacter pylori* infection, a well-established risk factor, continues to be a focal point in GC research, as does the impact of dietary habits, tobacco use, and familial predisposition [4]. Recent advances in molecular biology have illuminated the heterogeneity of gastric cancer, leading to the recognition of distinct subtypes that have different prognoses and therapeutic implications. The integration of genomics and precision medicine is shaping the development of targeted therapies and personalized treatment strategies for patients [ 5, 6] .

The *FANCA* gene is a vital component of the Fanconi anaemia (FA) pathway, a complex network of proteins responsible for mending DNA interstrand crosslinks (ICLs). These crosslinks arise when both strands of the DNA double helix are covalently linked, posing a significant threat to genomic stability. If left unrepaired, these crosslinks can lead to chromosomal abnormalities, cell death, and increased susceptibility to cancers [7]. Positioned on the long arm of chromosome 16 (16q24.3), the *FANCA* gene extends across a genomic region comprising multiple exons and introns. This gene encodes the FANCA protein, a large protein crucial to the FA core complex. This complex, consisting of various FA proteins, such as FANCB, FANCC, FANCE, FANCF, FANCG, FANCL, and others, is central to activating the FA pathway [ 8, 9] . The role of FANCA involves the assembly and stability of this core complex. The core complex is essential for the monoubiquitination of the FANCD2 and FANCI proteins, a critical step in FA pathway activation. The activated FANCD2-FANCI complex plays a central role in coordinating the repair of DNA interstrand crosslinks [10]. Mutations in the *FANCA* gene can lead to FA, resulting in a non-functional or less stable FANCA protein. This disruption affects the formation of the FA core complex, impairing the repair of DNA interstrand crosslinks and contributing to the phenotypic manifestations of FA. [11]. Individuals with biallelic mutations in FANCA may exhibit various clinical features, including bone marrow failure, congenital anomalies (such as skeletal abnormalities), and an elevated risk of cancer.

In the present study, we assessed the expression of FANCA in GC and its prognostic significance. We also investigated the impacts of FANCA on the proliferation, migration, and invasion of GC cells. Finally, we explored the effects of FANCA on the cell cycle pathway.

## Materials and Methods

### Bioinformatics analysis

Clinical characteristics of stomach cancer patients were derived from the Cancer Genome Atlas (TCGA) database. StringDB [12] was used to identify various genes that interact with the *FANCA* gene. Subsequently, the identified genes were subjected to Kyoto Encyclopedia of Genes and Genomes (KEGG) and Gene Ontology (GO) enrichment analysis using DAVID bioinformatics tools [13]. The analysis criteria included a minimum gene set of 5, a maximum gene set of 5000, and statistical significance defined by a *P* value <0.05 and a false discovery rate (FDR) <0.25. FANCA expression levels were categorized into two groups based on samples with high and low expression. The association between FANCA expression and genes related to the cell cycle pathway was investigated using Gene Set Enrichment Analysis (GSEA) v4.3.2 software.

### Patients and GC specimens

Between July 2015 and May 2017, we acquired 80 sets of formalin-fixed and paraffin-embedded human GC specimens, with ages ranging from 43 to 77 years. The specimens for this study were generously provided by the Northern Jiangsu People’s Hospital, School of Clinical Medicine, Yangzhou University (Yangzhou, China). Importantly, none of the patients underwent preoperative chemotherapy. The stomach cancer diagnoses were confirmed by two distinguished pathologists. The American Joint Committee on Cancer TNM staging system for GC (AJCC-8 TNM) was used to determine the disease stage. This project received approval from the Ethics Committee of Northern Jiangsu People’s Hospital, Yangzhou University’s School of Clinical Medicine, with approval number 2019 KY-022. Before participating in the trial, all subjects provided written informed consent.

### Immunohistochemistry

A tissue microarray (TMA) comprising samples from 80 patients with histologically confirmed GC and 80 control subjects was created. This TMA was generated using a previously method [14]. The tissue sections were deparaffinized with xylene, rehydrated in a series of graded alcohol solutions and citrate buffer, and then blocked with 3% hydrogen peroxide. Subsequently, the sections were subjected to incubation with a primary antibody against FANCA (1:100; 11975-1-AP; Proteintech, Chicago, USA), followed by incubation with a biotin-conjugated secondary antibody (SA1050; Boster, Wuhan, China). This was succeeded by further incubation with a streptavidin-peroxidase complex. For each slide, five high-power fields (400× magnification) were randomly selected, and images were captured. Protein expression scoring was performed by taking into account both the proportion of positive cells (scored as follows: 0, <5%; 1, 5%–25%; 2, 26%–50%; 3, 51%–75%; and 4, >75%) and the intensity of cell staining (scored as follows: 0, negative; 1, weak; 2, moderate; and 3, strong). The final score for each patient was determined as the sum of these two scores. Patients with a score less than 4 were categorized as having low FANCA expression, while those with a score greater than or equal to 4 were classified as having high FANCA expression.

### Cell culture and drug treatment

In this study, we utilized human GC cell lines (AGS, NCI-N87, HGC-27, and BGC-823) and the epithelial cell line GES-1. These cell lines were obtained from the Cell Bank of the Chinese Academy of Sciences (Shanghai, China). All the cells were cultured in RPMI 1640 medium (HyClone, Logan, USA) supplemented with 10% fetal bovine serum (FBS) from Life Technologies (Waltham, USA) and 1% penicillin/streptomycin (Life Technologies). The cells were cultured at 37°C in a humidified incubator with a 5% CO _2_ atmosphere. Palbociclib obtained from MCE (HY-50767; Monmouth Junction, USA) was dissolved in DMSO and introduced into the culture medium at the specified concentration. Cell exposure to the drug lasted for 2 days at 37°C, unless stated otherwise.

### Cell transfection

The siRNAs were obtained from GenePharma (Shanghai, China). Cells ware seeded in 24-well plates at 1×10 ^4^ cells per well and allowed to incubate for 12 h. The transfection of these cells was carried out using Lipofectamine® 2000 from Thermo Fisher (Waltham, USA). After a 2-day transfection period, the cells were collected for subsequent analysis. The sequences were as follows: siNC-sense 5′-UUCUCCGAACGUGUCACGUTT-3′; siNC-antisense 5′-ACGUGACACGUUCGGAGAATT-3′; FANCA-siRNA-sense 5′-GCUGAUGCUCUUUCAGAUATT-3′; and FANCA-siRNA-antisense 5′-UAUCUGAAAGAGCAUCAGCTT-3′. The effectiveness of transfection was assessed by western blot analysis.

To establish stable FANCA overexpression in AGS and BGC-823 gastric cancer cell lines, lentivirus was used. Two recombinant lentiviral vectors, a blank (VP007-CMV-MCS-3flag-EF1-puro) and a FANCA (VP007-CMV-MCS-3flag-EF1-puro), were constructed by General Boil (Shanghai, China). In a 24-well plate, 1×10 ^4^ cells were plated in each well 12 h prior to viral infection. Lentivirus was added to each well and incubated for 72 h, followed by puromycin selection to identify stable cell lines. The transfection efficiency was evaluated by western blot analysis.

### CCK-8 assay

Cell proliferation was measured by CCK-8 assay. Briefly, cells were seeded in a 96-well plate, with each well containing 1×10 ^3^ cells. At time intervals of 24, 48, 72, and 96 h, 10 μL of CCK-8 solution (Yeasen, Shanghai, China) was added to each well and then incubated for 2 h. The optical density (OD) of each well was measured at 450 nm using a microplate reader (SkanIt RE 7.0; Thermo Fisher Scientific).

### Colony formation assay

Cells were plated in 6-well plates at a concentration of 1×10 ^3^ cells per well. Fresh medium supplemented with 10% FBS was regularly added during the incubation period. After two weeks, the cells were fixed for 10 min using 4% paraformaldehyde, stained with a 0.1% crystal violet solution for 10 min, air-dried, and photographed, and the number of colonies was quantified using ImageJ v1.54d software. Each experiment was conducted in triplicate, with at least three repetitions.

### Wound healing assay

Wound-healing assays were conducted to evaluate the migratory ability of the cells. The transfected cells were placed in six-well plates and continuously incubated until a uniform monolayer of cells covered the bottom of the plate. A micropipette tip was then used to create a controlled scratch wound on the cell surface, which was performed slowly and uniformly. Subsequently, the cells were washed multiple times with PBS to eliminate any floating cells. The remaining cells were cultured in RPMI 1640 without FBS. Wound images were captured at 0, 24, and 48 h using an inverted microscope (CKX53; Olympus, Tokyo, China). These images were subjected to analysis using ImageJ software (v1.54d).

### Transwell assay

A Transwell assay was conducted to evaluate the migration and invasion abilities of the cells. After transfection, the cells were cultured in 6-well plates for 48 h before the experiments commenced. Subsequently, the cells were rinsed twice with PBS, trypsinized using a 0.25% trypsin cell digestive solution (Beyotime), centrifuged, and resuspended in serum-free RPMI 1640 medium, after which they were counted. Next, 200 μL of the cell suspension was placed into the upper chamber, which was either coated with matrix (BD Biocoat, Franklin Lakes, USA) or left without matrix (Corning, Corning, USA). Meanwhile, 500 μL of RPMI 1640 medium containing 10% FBS was added to the lower chamber. The Transwell chambers were then incubated in a cell culture incubator for 48 h. After incubation, the cells on the upper surface of the chamber were gently removed using a cotton swab. The cells on the lower surface of the chamber were fixed with 4% paraformaldehyde for 10 min, stained with a 0.1% crystal violet solution for 5 min, washed twice with PBS, left to dry, and images were captured using an inverted microscope (CKX53; Olympus). The images were subsequently analyzed using ImageJ software (v1.54d).

### Flow cytometry analysis

To assess the cell cycle distribution, flow cytometry was used. The cells were first rinsed twice with PBS and then treated with trypsin, followed by a 4-h exposure to 70% ethanol at 24°C. Subsequently, the cells were placed in 500 μL of a prepared PI staining solution (Yeasen), incubated at 37°C for 30 min, and detected with a flow cytometer (BD LSRFortessa; BD Biosciences, Franklin Lakes, USA ). The resulting data were analyzed using FlowJo software (v10.8.1).

### Quantitative real-time PCR (qRT-PCR)

RNA was extracted from tissues and cells using Trizol reagent (Vazyme, Nanjing, China). Subsequently, cDNA was generated with Hifair® III 1st Strand cDNA Synthesis SuperMix for qPCR (gDNA digester plus) (Yeasen). Quantitative real-time PCR was conducted using Hieff® qPCR SYBR Green Master Mix (High Rox Plus) (Yeasen) on a StepOne Plus Real-Time PCR System (Applied Biosystems, Foster City, USA). To determine the relative expression level of *FANCA* mRNA, normalization was performed with *GAPDH* as an endogenous control using the 2 ^−ΔΔCt^ method. The primer sequences used are as follows: for *FANCA*, forward: 5′-GCTTGAGGTAGAAGGTCCACTGTG-3′ and reverse: 5′-GCCTTGAGGCTTGATCCTGCAAAG-3′. For *GAPDH*, forward: 5′-ACGGATTTGGTCGTATTGGGCG-3′ and reverse: 5′-GCTCCTGGAAGATGGTGATGGG-3′.

### Western blot analysis

Cells were lysed using RIPA buffer obtained from Beyotime (Shanghai, China). After the protein concentration was determined, each sample containing 30 μg of protein was separated by SDS-PAGE and subsequently transferred to polyvinylidene difluoride membranes (Millipore, Billerica, USA) with a pore size of 0.45 μm. After three washes with TBST, the membranes were blocked with 5% skim milk for 2 h at room temperature. Next, the membranes were incubated with a primary antibody overnight at 4°C. After another three washes with TBST, the membranes were treated with a secondary antibody conjugated with horseradish peroxidase (1:10,000; ABclonal, Burlingame, USA) for 2 h at room temperature. An enhanced chemiluminescent substrate from NCM Biotech (Suzhou, China), was used to detect the signals. The primary antibodies used in this study included anti-FANCA (1:5000; Proteintech), anti-Cyclin D1 (1:1000; ZENBIO, Chengdu, China), anti-CDK4 (1:1000; ZENBIO), anti-CDK6 (1:1000; ZENBIO), anti-CDC6 (1:4000; Proteintech), anti-PCNA (1:3000; Affinity, Cincinnati, USA), anti-MCM3 (1:3000; Proteintech), anti-MCM4 (1:3000; Proteintech), and anti-RFC3 (1:3000; Proteintech) antibodies.

### Xenograft model

A total of 10 female BALB/c nude mice, aged 4 weeks with a weight range of 18–22 g, were procured from GemPharmatech (Nanjing, China). The mice were housed under controlled conditions in a pathogen-free laminar flow cabinet for the duration of the experiments. These conditions included a humidity of 30%–40%, a constant temperature of 25°C, a 12/12-h light/dark cycle, and unrestricted access to food and water. The animal experiments were carried out following ethical approval granted by the Ethics Committee for Animal Experiments at Yangzhou University (yzu-lcyxy-s036). The experimental protocols adhered to the Laboratory Animal Guidelines for Ethical Review of Animal Welfare [15]. Four-week-old female BALB/c nude mice were randomly assigned to two groups, namely, the vector and oe-FANCA groups, each containing 5 mice. These mice were subjected to isoflurane inhalation anesthesia (1%–2%). Approximately 1×10 ^6^ BGC-823 cells from stably transfected Vector/oe-FANCA strains resuspended in 100 μL of PBS were subcutaneously injected into the left armpit of each mouse. The health and behavior of the mice were monitored at 2-day intervals to detect any signs of eating or drinking difficulties, unrelieved pain, or distress without recovery. If a tumor reached a volume of 2000 mm ^3^, humane euthanasia was administered as an endpoint. The tumor volume (V) was calculated every week using the following formula: V=(width ^2^×length)/2. Four weeks postinoculation, all the mice were humanely euthanized through cervical dislocation under anesthesia. CO _2_ asphyxiation was used as the method of anesthesia, with CO _2_ introduced into the chamber at a rate of 40%–70% of the chamber volume per minute to minimize distress. Dilated pupils were observed to verify death, and subsequently, the tumors were removed and weighed.

### Statistical analysis

Data were analyzed using GraphPad Prism v8.4.3 software and presented as the mean±standard deviation. To assess the difference between two groups, Student’s *t* test was applied, while for comparing differences among multiple groups, Dunnett’s test within the framework of an ordinary one-way ANOVA was used. Statistical significance was determined at a significance level of *P*<0.05.

## Results

### FANCA expression is distinctly elevated in GC cells

**Figure 1:**
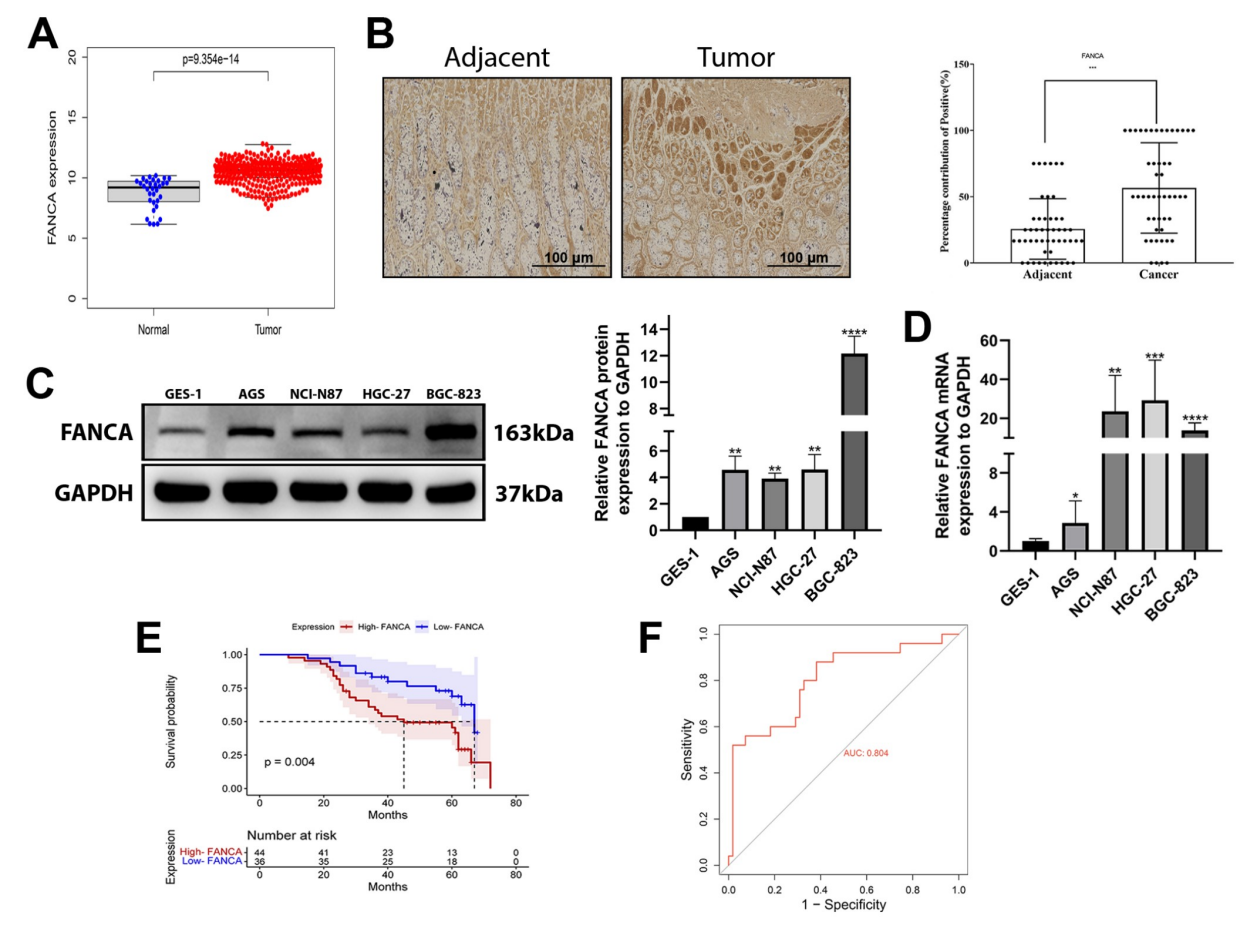
Figure 1 Elevated FANCA expression in GC cells (A) Comparison of FANCA expression between tumor tissues and adjacent tissues from the TCGA database ( P=9.354×10 –14). (B) IHC analysis of FANCA expression in 80 paired GC tissues and adjacent nontumor tissues. (C) Protein expression levels of FANCA in GC cell lines (AGS, NCI-N87, HGC-27, and BGC-823) and the normal gastric cell line GES-1, with GAPDH as a loading control (** P<0.01, and **** P<0.0001). (D) mRNA expression levels of FANCA in GC cell lines and the normal gastric cell line GES-1, with GAPDH as an internal control (* P<0.05, ** P<0.01, *** P<0.001, and **** P<0.0001). (E) Survival analysis of GC patients based on different FANCA expression levels. (F) ROC curve for FANCA expression, demonstrating excellent specificity and sensitivity with an AUC of 0.804.

An upregulation of FANCA expression was observed in the TCGA database when comparing tumor tissues to their corresponding normal tissues ( Figure 1A). Immunohistochemical analysis of 80 GC specimens indicated significant upregulation of FANCA in GC tissues ( Figure 1B). Furthermore, we performed cellular-level investigations. Western blot analysis and qRT-PCR of the cell lines revealed increased FANCA expression in four distinct GC cell lines (AGS, NCI-N87, HGC-27, and BGC-823) compared to the normal cell line GES-1 ( Figure 1C,D). Additionally, we explored the correlation between FANCA expression and various clinicopathological characteristics and revealed that increased FANCA expression is correlated with tumor size ( *P*=0.003), depth of invasion ( *P*=0.049), lymph node metastasis ( *P*=0.007), distant metastasis ( *P*=0.026), and TNM stage ( *P*=0.015). However, no significant association was detected between FANCA expression and age ( *P*=0.759), sex ( *P*=0.408), Lauren type ( *P*=0.797), degree of differentiation ( *P*=0.187), histological grade ( *P*=0.617), venous invasion ( *P*=0.866), or nerve invasion ( *P*=0.463) ( Table 1). To further investigate the link between FANCA expression and patient outcomes, we examined the overall survival (OS) of 80 GC patients. K-M analysis revealed a positive association between elevated FANCA protein expression and poor OS in GC patients ( *P*=0.004; Figure 1E). These findings suggested that FANCA levels are elevated in GC patients and that patients with high FANCA expression have an unfavourable prognosis. Subsequently, we employed receiver operating characteristic (ROC) analysis to assess the clinical diagnostic value of FANCA expression, which demonstrated excellent specificity and sensitivity with an area under the curve (AUC) of 0.804 ( Figure 1F).

**Table:** **Table 1** Associations between FANCA expression and clinicopatho-logical features of 80 patients with gastric cancer

Histopathological parameters	Total number ( *n*=80)	Expression level of FANCA	*P* value
		Low	High	
Age (years)				0.759
< 65	33	15	18	
≥ 65	47	23	24	
Sex				0.408
Male	51	26	25	
Female	29	12	17	
Tumor size (mm ^3^)				0.003
< 6	43	27	16	
≥ 6	37	11	26	
Lauren type				0.797
Intestinal type	73	35	38	
Diffuse type	7	4	3	
Depth of invasion				0.049
T1‒T2	29	18	11	
T3‒T4	51	20	31	
Lymphonodus metastasis				0.007
N0‒N1	40	25	15	
N2‒N3	40	13	27	
Distant metastases				0.026
Negative	58	32	26	
Positive	22	7	15	
TNM stage				0.015
I	27	14	13	
II	24	12	12	
III	17	8	9	
IV	12	5	7	
Degree of differentiation				0.187
Highly	29	14	15	
Moderately and poorly	51	17	34	
Histological grade				0.617
I	8	4	4	
II	38	20	18	
III	34	14	20	
Venous invasion				0.866
No	45	21	24	
Yes	35	17	18	
Nerve invasion				0.463
No	35	15	20	
Yes	45	23	22

### *FANCA* knockdown inhibits tumor cell proliferation, migration, and invasion

**Figure 2:**
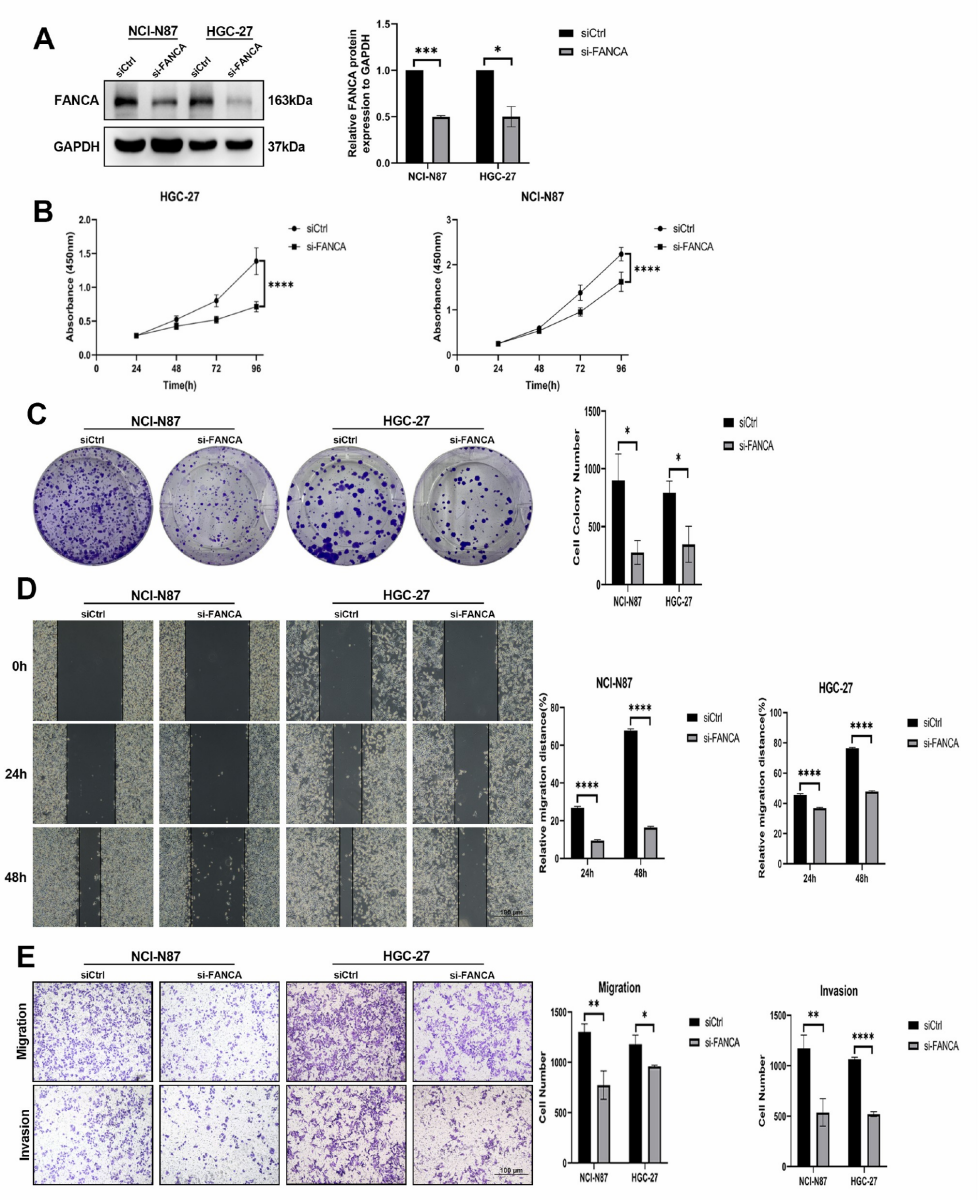
Figure 2 Suppression of FANCA impedes tumor cell proliferation, migration, and invasion (A) The efficacy of FANCA knockdown in NCI-N87 and HGC-27 cells transfected with si-FANCA was evaluated by western blot analysis. siCtrl denotes the negative control. (B,C) The viability and proliferative capacity of NCI-N87 and HGC-27 cells transfected with siCtrl or si-FANCA were determined using CCK8 and colony formation assays. (D) Wound healing assay assessing the motility of NCI-N87 and HGC-27 cells transfected with siCtrl or si-FANCA. (E) Transwell assays were used to quantify the number of migrated and invaded NCI-N87 and HGC-27 cells transfected with siCtrl or si-FANCA. * P<0.05, ** P<0.01, *** P<0.001, and **** P<0.0001.

To investigate the impact of reducing FANCA expression on the growth of GC cells, we used RNAoligo-mediated siRNA to downregulate endogenous FANCA levels in highly expressing NCI-N87 and HGC-27 cells. This knockdown efficiency was verified by western blot analysis ( Figure 2A). To assess the influence of FANCA on the proliferation of NCI-N87 and HGC-27 cells, CCK8 and colony formation assays were conducted. The results revealed a significant reduction in cell proliferation when *FANCA* was knocked down compared to that in the control group ( Figure 2B,C). Next, we examined the migratory and invasive abilities of NCI-N87 and HGC-27 cells following *FANCA* knockdown. The results from wound-healing and transwell assays demonstrated a substantial decrease in migration and invasion in the si-FANCA group compared to those in the siCtrl group for both NCI-N87 and HGC-27 cells ( Figure 2D,E). There was no significant difference in growth, migration, or invasion between the si-FANCA NCI-N87 and HGC-27 groups.

### *FANCA* overexpression promotes tumor cell proliferation, migration, and invasion

**Figure 3:**
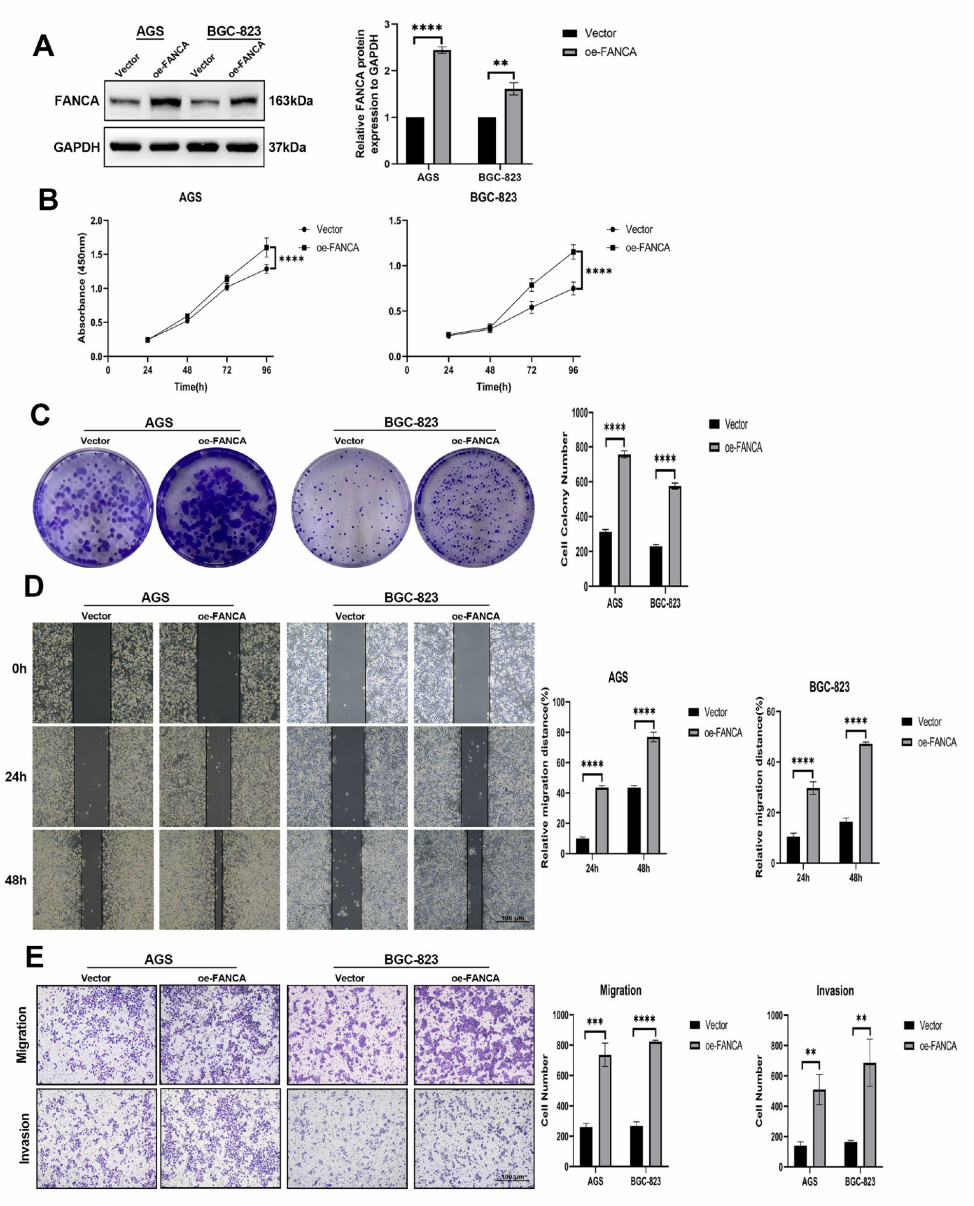
Figure 3 Elevated FANCA expression enhances tumor cell proliferation, migration, and invasion (A) The effectiveness of upregulating FANCA level in AGS and BGC-823 cells transfected with oe-FANCA was assessed via western blot abalysis. The blank is denoted as Vector, while cells with FANCA overexpression are labelled as oe-FANCA. (B,C) The viability and proliferative capacity of AGS and BGC-823 cells transfected with vector or oe-FANCA were determined using CCK8 and colony formation assays. (D) Wound healing assay evaluating the motility of AGS and BGC-823 cells transfected with vector or oe-FANCA. (E) Transwell assays were used to quantify the number of migrated and invaded AGS and BGC-823 cells transfected with vector or oe-FANCA. ** P<0.01, *** P<0.001, and **** P<0.0001.

To assess whether elevated level of FANCA contribute to the enhanced proliferation and invasion of GC cells, we employed lentiviral transfection to establish continuous overexpression of FANCA in AGS and BGC-823 cells ( Figure 3A). Upon transfection with *FANCA*, there was a significant increase in its protein level in AGS and BGC-823 cells ( Figure 3A,B). Subsequent CCK-8 and colony formation assays demonstrated a substantial increase in cell proliferation due to the upregulation of FANCA ( Figure 3C,D). Furthermore, the results of the wound healing and Transwell experiments revealed a marked increase in cell migration and invasion capacity as a result of FANCA upregulation ( Figure 3E,F). Notably, there was no notable difference in growth, migration, or invasion between the oe-FANCA AGS and BGC-823 groups.

### FANCA is involved in the transition from G1 to S phase

**Figure 4:**
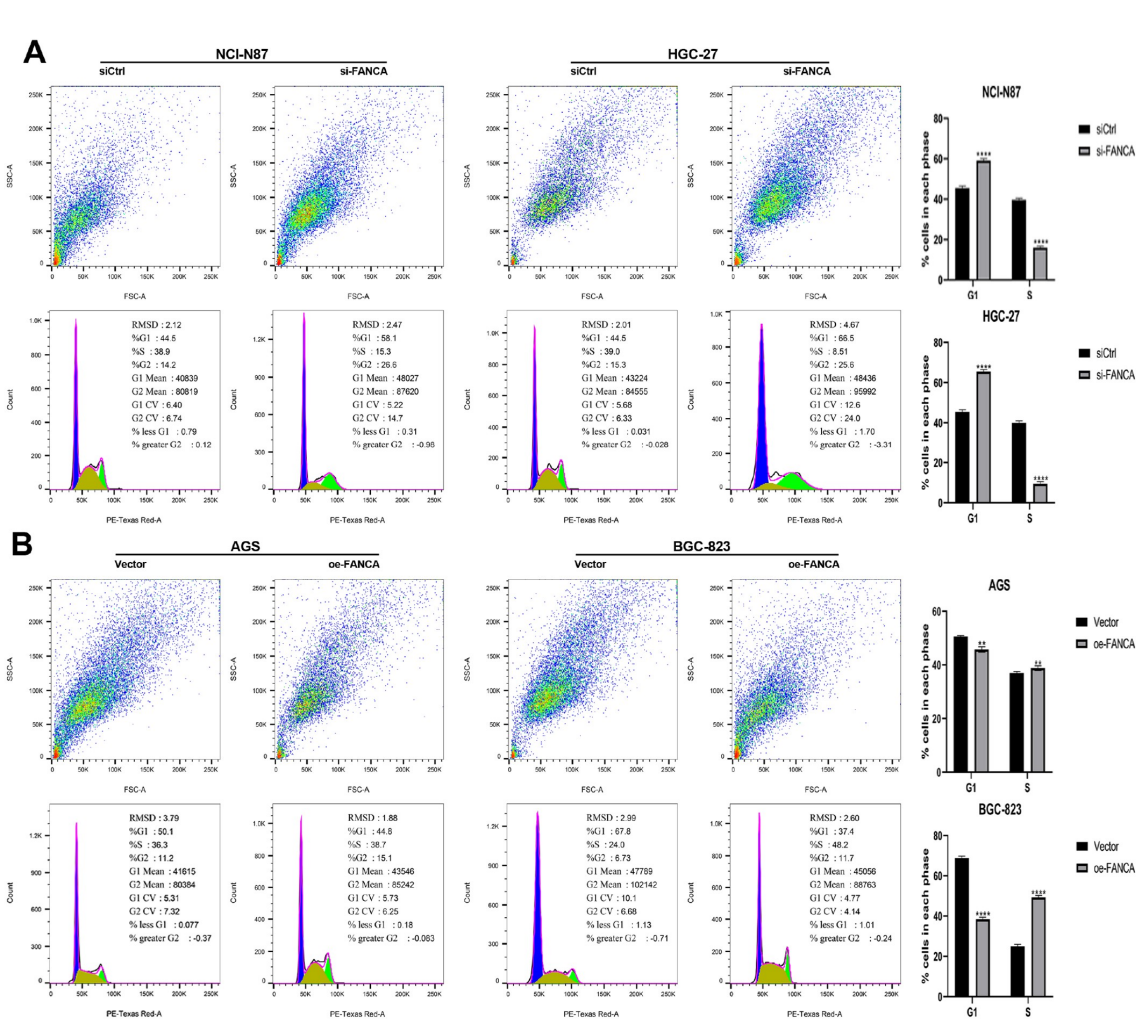
Figure 4 FANCA contributes to the transition from G1 to S phase in the cell cycle (A) The impact of FANCA knockdown on the cell cycle of NCI-N87 and HGC-27 cells was assessed using a cell cycle and apoptosis analysis kit. (B) The influence of FANCA overexpression on the G1 to S phase transition in AGS and BGC-823 cells was examined using a cell cycle and apoptosis analysis kit. ** P<0.01, and **** P<0.0001.

To provide a concise overview of the experimental objectives, our primary aim was to investigate the role of the *FANCA* gene in the cell cycle, with a specific focus on its involvement in the G1/S phase transition. Using flow cytometry, we sought to examine whether manipulating FANCA expression impacts cell cycle progression in GC cells. The results showed that when FANCA level was reduced in NCI-N87 and HGC-27 cells, notable prevention of cell cycle arrest in the G1 phase was observed ( Figure 4A). Conversely, increase in FANCA expression in AGS and BGC-823 cells facilitated G1 phase cell cycle arrest ( Figure 4B). These findings contribute valuable insights into the regulatory role of FANCA in orchestrating cell cycle dynamics, particularly at the G1/S phase transition, within the context of GC cells.

### Overexpression of FANCA promotes tumor progression *in vivo*

**Figure 5:**
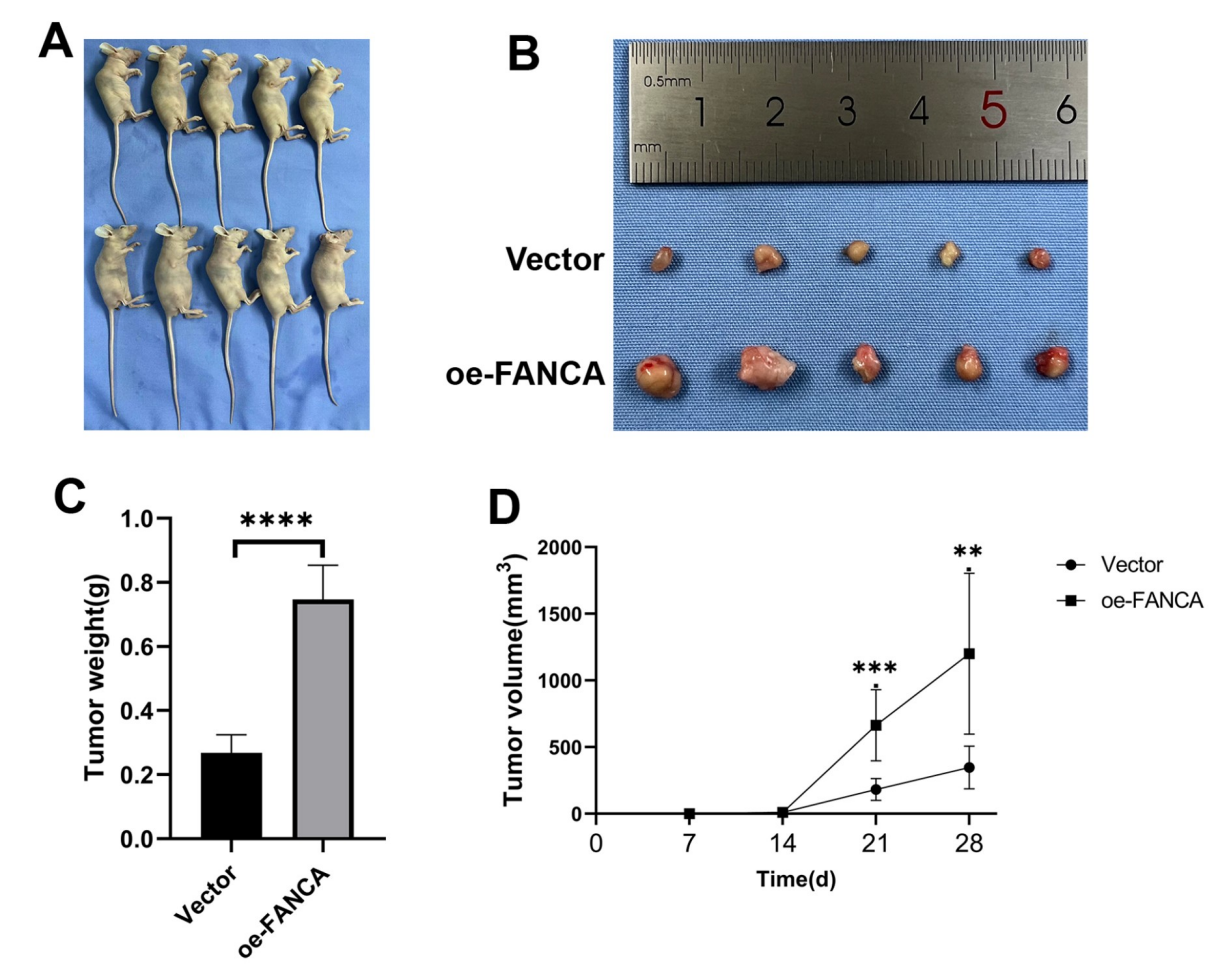
Figure 5 FANCA enhances tumor progression *in vivo* (A) Xenograft models were established in nude mice using BGC-823 cells transfected with vector ( n=5) or oe-FANCA ( n=5). (B) Images of subcutaneous tumors. (C) Weights of the subcutaneous tumors. (D) Growth curves of subcutaneous tumors in nude mice. ** P<0.01, *** P<0.001, and **** P<0.0001.

A xenograft tumor model was used to further investigate the impact of FANCA *in vivo*. Nude mice received injections of BGC-823 cells that had been consistently transfected with either the vector or the oe-FANCA lentivirus. The growth of xenograft tumors was monitored weekly following the injection. Compared with the vector group, the FANCA overexpression group exhibited a considerable increase in tumor size ( Figure 5A,B). The largest tumor volume observed was 0.906 mm ^3^ in the oe-FANCA group, while the vector group showed a smaller tumor volume of 0.312 mm ^3^ 4 weeks after the mice were euthanized ( Figure 5C). Subsequently, the tumors from both groups were weighed, revealing a significantly greater average weight in the oe-FANCA group than in the vector group (1200±0.130 g vs 348±0.039 g; *P*<0.05) ( Figure 5D). These findings indicated that the overexpression of FANCA actively promotes the *in vivo* growth of GC xenograft tumors.

### FANCA exerts its biological effects through the cell cycle signaling pathway

**Figure 6:**
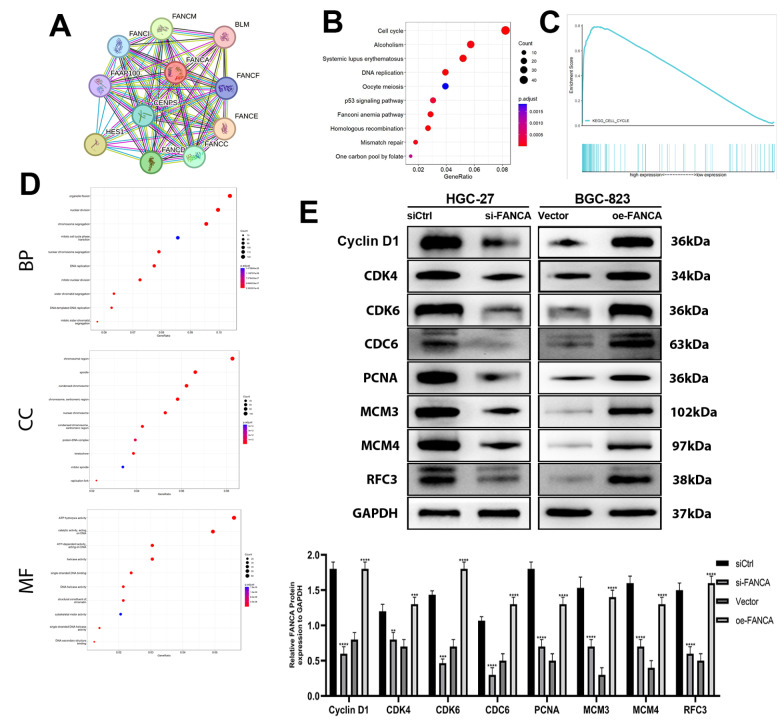
Figure 6 FANCA enhances activation of the cell cycle pathway (A) Genes displaying significant interactions with FANCA. (B) Enrichment analysis of interacting genes using the Kyoto Encyclopedia of Genes and Genomes (KEGG). (C) Gene Set Enrichment Analysis (GSEA) assessing the correlation between FANCA expression and the gene signature associated with the cell cycle pathway. (D) Enrichment analysis of interacting genes based on Gene Ontology (GO) analysis. (E) Western blot analysis was utilized to evaluate the protein levels of Cyclin D1, CDK4, CDK6, CDC6, PCNA, MCM3, MCM4, and RFC3 in HGC-27 and BGC-823 cells following transfection with siCtrl, si-FANCA, vector, or oe-FANCA. ** P<0.01, *** P<0.001, and **** P<0.0001.

Stringdb revealed 10 genes that interact significantly with the *FANCA* gene ( Figure 6A). The 10 genes that interact with *FANCA* are *FANCI*, *FANCM*, *BLM*, *FAAP100*, *FANCF*, *CENPS*, *FANCE*, *HES1*, *FANCD*, and *FANCC*. According to KEGG analysis, the genes interacting with each other primarily participate in pathways related to the cell cycle, alcoholism, systemic lupus erythematosus, and similar processes ( Figure 6B). In terms of biological processes (BP), the interacting genes are implicated in activities such as organelle fission, nuclear division, chromosome segregation, and related processes ( Figure 6D). Molecular function (MF) analysis revealed that the interacting genes are engaged in functions such as ATP hydrolysis and catalytic activity, particularly by acting on DNA ( Figure 6D). Concerning cellular components (CCs), the interacting genes contribute to structures such as chromosomal regions, spindles, and condensed chromosomes ( Figure 6D). Furthermore, the mRNA expression of FANCA in gastric cancer patients was categorized into high and low-expression groups based on the median value. Gene set enrichment analysis (GSEA) demonstrated that elevated FANCA expression activated the cell cycle pathway ( Figure 6C).

The cell cycle pathway is widely recognized for fostering uncontrolled cell growth, invasion into neighboring tissues, and in advanced stages, metastasis. It is a crucial pathway implicated in the initiation and progression of GC [16]. Consequently, our investigation aimed to ascertain whether FANCA could stimulate the cell cycle pathway to facilitate tumor development. Protein imprinting analysis revealed decreased levels of Cyclin D1, CDK4, CDK6, CDC6, PCNA, MCM3, MCM4, and RFC3 in HGC-27 cells transfected with si-FANCA, while the opposite trend was observed in oe-FANCA-transfected BGC-823 cells ( Figure 6E). These findings suggest that FANCA potentially promotes tumor development by activating the cell cycle pathway.

**Figure 7:**
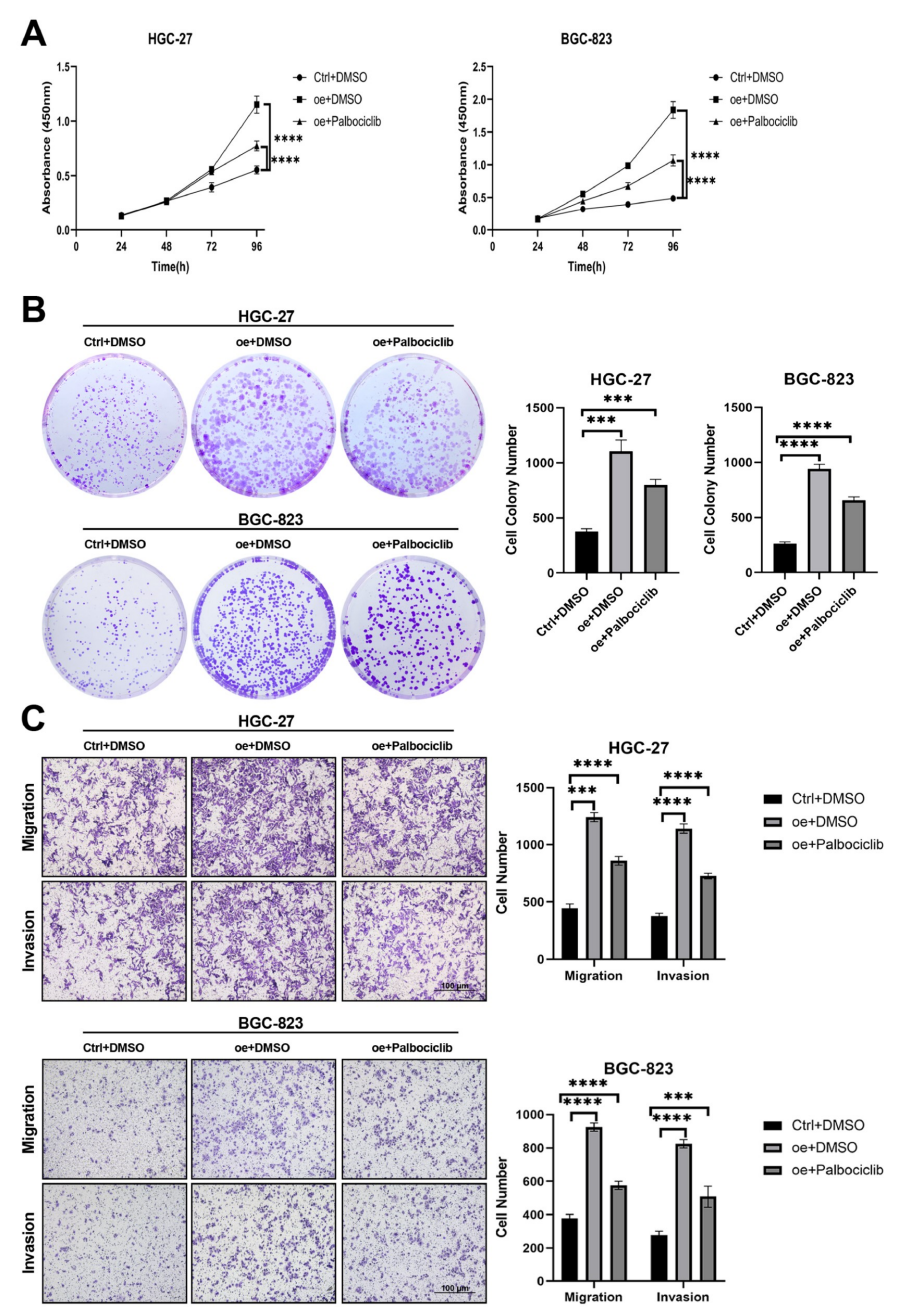
Figure 7 FANCA exerts its biological functions through the cell cycle pathway (A,B) Cell viability and proliferation in HGC-27 and BGC-823 cells subjected to different treatments were assessed using CCK-8 and colony formation assays: (i) Ctrl+DMSO, (ii) oe+DMSO, and (iii) oe+Palbociclib. (C) Transwell assays were conducted to measure the migration and invasion abilities of HGC-27 and BGC-823 cells without treatment: (i) Ctrl+ DMSO, (ii) oe+DMSO, and (iii) oe+palbociclib. Palbociclib was dissolved in a 10 mM stock solution in DMSO, and the concentration of palbociclib was 2 mM, with 0.02% DMSO used as a negative control. *** P<0.001, and **** P<0.0001.

To further validate the involvement of the cell cycle, we used a CDK inhibitor (palbociclib). HGC-27 and BGC-823 cells, characterized by low FANCA expression, were treated with oe-FANCA and palbociclib. The results indicated that FANCA overexpression significantly increased cell proliferation, migration, and invasion. Conversely, palbociclib treatment inhibited these biological behaviors ( Figure 7A‒C). These data strongly imply that FANCA may enhance cell proliferation, migration, and invasion by activating the cell cycle pathway.

## Discussion

The primary association of the *FANCA* gene is with FA, a rare genetic disorder characterized by bone marrow failure, congenital abnormalities, and an increased risk of cancer. The increased cancer risk in individuals with FA is diverse and includes various types of malignancies, such as acute myeloid leukemia (AML), squamous cell carcinomas, and solid tumors affecting different organs [ 10, 17, 18] . Nevertheless, the impact of FANCA on the progression of GC and its underlying mechanisms remain uncertain. The present investigation revealed a notable upregulation of FANCA level in cell lines associated with GC. FANCA, in turn, facilitates the advancement of GC cells by modulating malignancies such as proliferation, migration, and invasion, indicating its potential as a promoter of tumor progression in GC.

Earlier research conducted by Lach *et al*. [19] revealed a correlation between elevated FANCA expression and the metastatic stage of esophageal cancer. Our results align well with their findings, indicating that the suppression of FANCA hinders the proliferation, migration, and invasion of GC cells. Furthermore, *in vivo* experiments demonstrated that FANCA plays a promotional role in the tumor progression of GC.

Understanding the dynamics of cellular behavior, particularly in diseases such as cancer, centers on the intricate relationship between cell cycle regulation and invasion. The cell cycle governs cell division through a highly regulated process, and invasion, the ability of cells to infiltrate surrounding tissues, shares connections influencing cellular fate [20]. Dysregulation of the cell cycle, marked by aberrant control points and disrupted checkpoints, is implicated in various pathological conditions. In the context of invasion, these disruptions significantly impact cellular behavior. Uncontrolled cell proliferation resulted from cell cycle dysregulation may contribute to enhanced invasion potential. Overexpression or mutation of key regulators, such as cyclins and cyclin-dependent kinases (CDKs), leads to sustained cell division and evasion of growth control mechanisms, fostering an environment conducive to invasion [21]. In cancer, where these relationships are particularly relevant, understanding the interplay between cell cycle dysregulation and invasion is paramount. Tumor cells with altered cell cycle machinery often exhibit increased invasive potential, facilitating metastasis to distant organs. This phenomenon underscores the clinical significance of unravelling the molecular intricacies connecting cell cycle regulation and invasion [22]. Dysregulation of the G1/S phase cell cycle is often associated with uncontrolled cell proliferation, a hallmark of cancer. The complex interplay between cell cycle proteins and CDKs regulates the intricacies of the cell cycle [23]. In this study, we found that the expression levels of metastasis markers were affected by changes in FANCA expression. Downregulation of FANCA level decreased the expressions of CDK4 and CDK6, while upregulation of FANCA level had the opposite effect. In addition, we found that the expression levels of Cyclin D1, CDC6, PCNA, MEM3, MCM4, and RFC3 decreased when *FANCA* was knocked down, while upregulation of FANCA showed the opposite trend. The G1/S phase transition is indeed a critical checkpoint in the cell cycle, and dysregulation at this point is frequently associated with cancer [24]. Our study demonstrated that FANCA downregulation decreased the proportion of cells in the G1/S phase of the cell cycle. In recent investigations, various CDK inhibitors, including palbociclib, ribociclib, and abemaciclib, were examined for their potential application in multiple tumors [ 25‒ 27] . CDKs, a family of enzymes that function in conjunction with their regulatory partners known as cyclins, govern the progression of the cell cycle [28]. To explore this relationship, we used a CDK inhibitor for comparison with FANCA overexpression in a functional recovery assay. Our findings indicate that the CDK inhibitor palbociclib significantly hinders the enhancing impact of FANCA upregulation on cell growth, migration, and invasion. These results imply that FANCA might stimulate cell proliferation, migration, and invasion by activating the cell cycle pathway.

Nevertheless, our research has certain limitations. To substantiate the biological role of FANCA in GC, additional *in vivo* experiments are essential. In the future, further experiments are warranted to provide a more detailed understanding of the involvement of FANCA in GC.

In conclusion, our findings suggest that FANCA is upregulated in GC. The elevated expression of FANCA appears to be strongly linked to the malignant biological characteristics of GC, suggesting a potential oncogenic role for FANCA through the G1/S phase cell cycle pathway in GC. This study shows promise for identifying potential markers and target genes for the treatment of gastric cancer.
